# Fall Detection System-Based Posture-Recognition for Indoor Environments

**DOI:** 10.3390/jimaging7030042

**Published:** 2021-02-26

**Authors:** Abderrazak Iazzi, Mohammed Rziza, Rachid Oulad Haj Thami

**Affiliations:** 1 LRIT, Raba IT Center, Faculty of Sciences, Mohammed V University in Rabat, Rabat B.P. 1014, Morocco; mohammed.rziza@gmail.com; 2 ADMIR LAB, IRDA, Rabat IT Center, ENSIAS, Mohammed V University in Rabat, Rabat B.P. 1014, Morocco; rachid.ouladhajthami@gmail.com

**Keywords:** fall detection, human posture recognition, classification, background subtraction, features extraction, video surveillance

## Abstract

The majority of the senior population lives alone at home. Falls can cause serious injuries, such as fractures or head injuries. These injuries can be an obstacle for a person to move around and normally practice his daily activities. Some of these injuries can lead to a risk of death if not handled urgently. In this paper, we propose a fall detection system for elderly people based on their postures. The postures are recognized from the human silhouette which is an advantage to preserve the privacy of the elderly. The effectiveness of our approach is demonstrated on two well-known datasets for human posture classification and three public datasets for fall detection, using a Support-Vector Machine (SVM) classifier. The experimental results show that our method can not only achieves a high fall detection rate but also a low false detection.

## 1. Introduction

The study in [1] shows that millions of elderly people—those aged 65 and over—fall each year. One in four elderly people falls, but less than half talk to their doctor. Successive falls usually come because of the first fall. Several factors cause falls, such as lower-body weakness, vision problems, difficulty walking and balancing, and the use of medicines. In order to live in a well-secured environment, researchers have proposed numerous systems to reduce the risk of falls for these elderly people.

Recently, many studies, approaches and applications were proposed for fall detection [2,3,4,5]. A recent one was proposed in [6], where the authors discuss the taxonomy of fall detection and the availability of fall data. According to sensor technology developed up to date, each proposed system is classified into two categrories: wearable-based method and camera-based method.

Fall detection systems based on worn sensors are the most commercialized systems that are basically electronic devices that need to be worn by the elderly, or put into his pocket or his clothes. They have many features that can be used to detect a fall. Such sensors are worn on garments with strain sensors to recognize upper-body posture [7]. Using the trixial-accelerometer mounted on the waist of the body in order to classify human movement status [8]. The manual help button is also used for sending for help after falling. However, even if they have many advantages, these sensors need to change their source of power periodically. On the other hand, they require to wear by humans during their Activities of Daily Life (ADL). The help button is useless if the person is unconscious after falling. For this purpose, it can be a source of inconvenience for them.

By comparing computer vision with other methods, it is found that computer vision presents a new solution to overcome the drawbacks of the previous methods. The main advantage is that there is no interaction with an elderly person, indeed, he does not need to wear anything. Moreover, it is able to give information for tracking and identifying several types of his activities as falls. With this method, we could also send an alarm signal with a short video clip as further confirmation of whether an elderly person has fallen. In the real-world environment, the fall detection is very challenging due to pose changes, light changing, shadows, illumination, occlusions, etc. To overcome these uncontrolled conditions, several fall detection approaches are proposed.

The remainder of this paper is organized as follows: in Section 2, the related works of computer vision for fall detection are presented. Section 3 details our proposed approach in which the human posture recognition combined with floor information for fall detection. Then, the experimental results and discussion of the proposed scheme are presented in Section 4. Finally, we give a general conclusion and we discuss some possible future works in Section 5.

## 2. Related Work

In recent years, visual-based fall detection systems have been used in many works. Many approaches based on computer vision have been proposed for fall detection. According to litterature, they can be classified as thresholding based methods [9,10,11,12,13,14,15] and machine learning based methods [16,17,18,19,20,21]. The first technique to detect a fall is by extracting different features and setting a threshold. If the extracted features are changed during a certain fixed interval, and they are larger/lower than the defined threshold, then the fall will be detected.

Detection of falls can be based on the analysis of the person’s posture. The main concept is to recognize some abnormal posture of the person such as bending, sitting and lying, and then use some characteristics to check for the occurrence of the fall. In [12], the authors proposed an algorithm of fall detection in which three different states are identified: fall prediction, fall detection, and fall verification state. In the first state, the posture of the person that is tracked is identified. Then, a prediction of the future posture is operated and this is compared to abnormal events similar to falls. The verification step comes after confirming the fall. The person’s posture is represented by four features: the occupied area, the person’s height, the person’s density, and the bounding box aspect ratio. The fall event is triggered if the posture is classified as a lie or sit on the floor for a long time. This detection should be confirmed based on a set of rules, the most important among them being the ability of the person to autonomously recover within 1 min. Hung et al. [13,14] proposed combine two orthogonal cameras to detect the falls incidents. Two features, the person’s height and occupied area, are estimated and used to distinguish three human postures (e.g., standing, sitting and lying). Based on some well-tuned thresholds, the fall incidents can be inferred from the time-series analysis of human pose transition. The dataset presented [22] was used to evaluate the algorithm based on videos recorded by camera 2 and 5.

Matilainen et al. [23] proposed a body part segmentation (BPS) for unusual activity recognition in noisy environments. The BPS is used to estimate the similarity of the current pose to the poses acquired in the training phase. For testing their algorithm, they consider only two normal activities such as Walking and Sitting while any other activity, including falling, is classified as unusual activity. The BPS algorithm uses the HMM and GMM classifiers which are trained for each body based on shape context features. In the proposed solution, three training sequences were used to represent examples of walking and falling. By the statistical analysis of the training sequences values of the thresholds are devised so as to enable the detection of unusual poses. They also proposed to use a majority voting over a large number of consecutive decisions for two reasons. First, the actions occur over a period of time. Second, to correct the effect of having several decisions from every single frame. However, this method is not helpful because it is based on a minimalist set of activities for training and the difficulty of choosing optimal threshold values.

Another rule-based fall detection method, presented in [15], relies on the use of a sliding window to detect a fall (SW-FDM) based on human postures. The proposed work distinguished between a real fall, lying and long-lie postures by using a predefined threshold. The experiments showed that the proposed SW-FDM is excellent in rule-based fall detection, also it is faster in processing time and low memory usage.

The previous methods are not considered universal fall detection systems because the thresholds should be adapted for every change as the position of the camera or the environment. In order to avoid these limitations, researchers have been moving their attention to machine learning techniques that can provide a general system for fall detection. In [18,19], the authors proposed a fall detection system using the semi-supervised method to detect an abnormal posture event and using the thresholding rules for a final decision based on movement analysis. The SVM classifier uses several features to describe the posture of the person, including the Angle and Ratio of a-axis and b-axis extracted from the ellipse, the shape-context histogram, and the person’s position. The Motion-energy of the image (MEI) is used to analyze the movement of the person. The fall is detected when the posture of the person is classified as lying or bending and the person’s velocity exceeds a pre-defined threshold. The experiments showed good results where they obtained 3% error rate which is lower than the results presented in [24].

The work in [20] presents a new fall detection system based on posture variation. The main idea is to use the Normalized Directional Histogram (NDH) extracted from the ellipse of the body region. 12 local features and 8 global features are extracted from the NDH. Then, an SVM classifier is used to predict, in each frame, the human pose. The fall-like accident is detected by counting the occurrences of lying postures in a short temporal window. The fall event is determined based on motionless verification after conducting majority voting. The system is evaluated on the dataset presented in [22] and achieves an accuracy up to 97.1%. However, in both solutions presented in [20,25] motion information is not considered, although this could reduce the rate of false alarm. The shape information is also used in [16] where the authors computed occupancy areas around the body’s gravity center, extracted their angles, and fed them into various classifiers, among them, the Support vector machine (SVM) which gives the best results. The same work has been improved in [26], where they combined the generalized likelihood ratio (GLR) and SVM. Only spatial information is not enough, so combining spatial features and temporal features has been used in [27]. Two stages of SVM classifier are used to detect a fall from normal activity. The advantage of this method is that it does not require a foreground technique to yield accurate results.

Using only 2D information may have drawbacks for fall detection. For this purpose, The authors in [21] exploit the Kinect camera for extracting 3d information. The Kinect camera provides 25 skeleton joints that are used to represent an activity. The system describes an activity by using a set of few basic postures extracted by means of the X-means clustering algorithm. A multi-class SVM classifier is used to discriminate the different postures. Another interesting approach has been proposed in [25] based on the silhouette volume orientation (SOV). SOV is a descriptor of shape that has been defined to represent human actions and also to classify falls. It offers an important classification accuracy and can lead to better results when combined with modeling tools like Bag-of-Words and the Bayesian Naive classifier. The system is evaluated on the dataset presented in [28]. The proposed system scores 91.89% accuracy that is lower than some previously discussed systems, yet being higher than the accuracy of the method proposed in [29]. Other work [30] focuses on obtaining the key points of the human body using Yolo and OpenPose algorithm. Then, they extracted two types of features named falling-state and fallen-state. The falling-state is mainly based on the speed change of the human body part. The fallen-state features are the human body key points and external ellipse. They combined Multi-Layer Perception (MLP) and Random-Forest (RF) for classification to obtain the fall detection results.

The other type of fall detection system is based on deep learning. The limitation of traditional machine learning are overcome by deep learning which has been applied in several application fields (e.g., natural language processing, object detection/classification, human actions). Therefore, recent works are led to use deep learning for fall detection. Feng P. et al. [17] use a deep belief network (DBN) for human posture classification. First, they extracted human silhouettes from video frames in order to train DBN for posture classification, then, they adopted a rule-based method for fall confirmation. The fall is confirmed when the lying posture events continue to occur for longer than 30 s. In [31], the authors used the Convolution Neural Network (CNN) for posture classification. The results obtained by these two methods were conducted on the same dataset [17], and CNN achieved higher accuracy than DBN. The success of applying deep learning in many other fields could be justified by the fact that the learning systems were conducted on real video data life. However, it is not for fall detection field because there are a few fall dataset for elderly that is not publically available. The existing datasets are only simulations of a young person which is totally different for an elderly and sick person and, therefore, classifications based on these datasets are capable of incorrectly detecting the fall of an elderly person in real life. Thus, using deep learning for fall detection based on computer vision is still limited.

Inspired by the promising results of the previously discussed methods, we propose two major contributions. First, we propose a new shape descriptor for human posture recognition. The robustness of our shape descriptor is evaluated on different datasets using different scenarios and comparisons against the approach cited above in our state of the art. By exploiting the result of posture recognition, we propose an effective algorithm for fall detection to insure that our posture recognition leads to improving fall detection accuracy and reducing false alarms.

## 3. Our Approach

**Figure 1:**
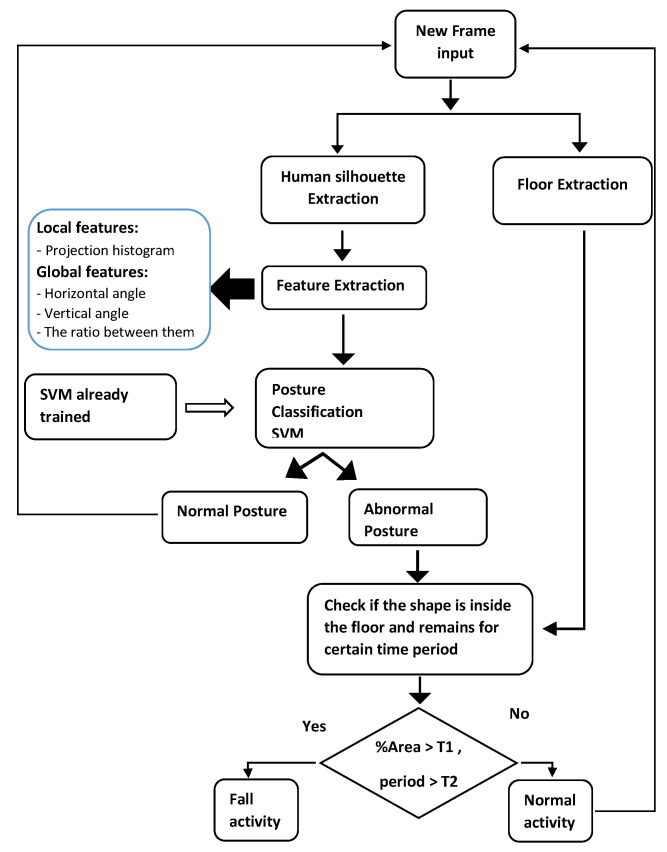
Main component of the fall detection approach.

This section presents the proposed approach. Figure 1 presents the whole fall detection system component. Our approach is composed of three phases. The first phase is for extracting the human silhouette from frames of the input video. The second phase is for extracting local and global features through the human silhouette. These features are combined and used for posture classification. By using our classifier, we distinguish between normal posture and abnormal posture. As for post-processing, we set four rules to validate if the current activity could be classified as fall or normal activity.

### 3.1. Background Subtraction

The monitoring systems based on computer vision consist of moving and static detection, video tracking to understand the events that occur in the scene. In our case, we are interested in detecting and extracting a moving person from the background, which is the most challenging task in fall detection systems-based computer vision. According to the literature, the common way to discriminate moving objects from the background is by using Background Subtraction (BS). Currently, many algorithms based BS are proposed, these include Gaussian Mixture Model (GMM) [32], Approximated Median Filter (AMF) [33] and CodeBook model (CB) [34].

For detecting a moving person in a video sequence, the algorithms should take into account some difficulties. The shape’s size of the human body is changing when the camera is far or is close to the human. The color and texture could be affected by shadows or when the living room is ambient light. For this reason, we will consider the Codebook method [34] for its advantage and robustness (generally) to detect and remove the shadows. The comparison results of these three algorithms could be found in [18].

Initially, our task consists to detect one moving object, the elderly, in the video sequence. The camera used is RGB and is installed two meters high in the living room. The background generally is static but if there is some moving furniture, it should be taken into account.

The CB algorithm is a pixel-based approach and it is composed of two phases: training phase (background model) where the algorithm constructs the codebook for each pixel of first *N* frames in a video; then, this codebook is used for background subtraction purpose.

**Figure 2:**
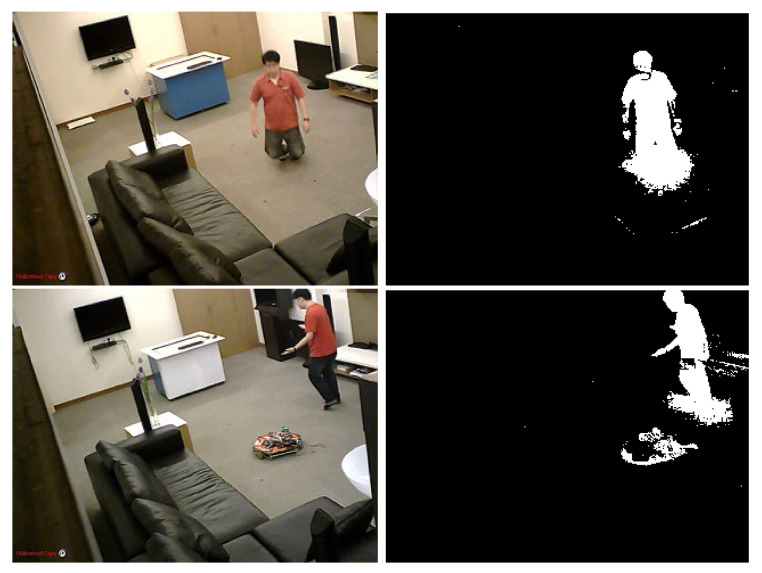
Codebook background subtraction result.

Generally, the result obtained by the CB algorithm is not perfect and it needs to be improved in order to get the human’s silhouette more accurately. In Figure 2, we show an example of two problems; first, many noisy pixels region which can be produced by shadows. Second, the movement of furniture can be detected as a moving object, then, the other pixels region is taken as a foreground object. These two problems will extremely deteriorate the result of body extraction. As a solution to this, we propose to add a post-processing step.

In the beginning of the post-processing step, we are interested in detecting and removing the shadows from the foreground based on the method proposed in [35]. We estimate the shadows using gradient information and HSV color, then we classify the shadow pixels based on pre-defined thresholds. For each new frame, we apply this step to remove the shadows without updating the background model because the shadow is an active object.

To detect shadow pixels, we first compare the HSV colors of the current frame *F* with their HSV color in reference frame *R*. HSV color space was used because of its advantages to separate chromaticity and intensity. Then, the gradient information is used to refine these shadow pixels.

The pre-processing operation step was used where we normalized the *V* component in HSV-color space to improve the contrast between shadow and non-shadow regions. Let I=(H,S,V) the original image where H,S, and *V* are its channel components. The *V* channel is normalized as follow:

(1) $$V_{n}=\frac{V-min(V)}{max(V)-min(V)},$$

where $V_{n}$ is the normalized *V* component and *V* is the original component of the image *I*. As a result, the normalized original image *I* obtained is as follow $I_{n}=\left(H,S,V_{n}\right)$.

To classify pixels to non-shadow/shadow pixel, we based on these rules as follow:

(2) $$\left\{\begin{array}{c} \alpha \leq \left(\frac{F_{p}^{V}}{R_{p}^{V}}\right)\leq \beta ,and\\ \left(F_{p}^{S}-R_{p}^{S}\right)\leq \tau_{S},and\\ \left|F_{p}^{H}-R_{p}^{H}\right|\leq \tau_{H} \end{array}\right.$$

where $\left(F_{p}^{H},F_{p}^{S},F_{p}^{V}\right)$ and $\left(R_{p}^{H},R_{p}^{S},R_{p}^{V}\right)$ are correspond to values of pixel p(i,j) in current Frame *F* and Reference frame *R* respectively; $\beta ,\tau_{S}$ and $\tau_{H}$ refer to thresholds used to detect shadow pixels. The range and optimized values of these thresholds are presented in Table 1.

After detecting all shadow pixels using HSV color, we extract all connected component Blobs (*B*) consisted by these shadow pixels. The gradient magnitude $\left|\nabla_{p}\right|$ and gradient direction $\theta_{p}$ are computed at each shadow pixels p(i,j) from (*B*) blob. Then, we only extract the significant gradient pixels that are higher than gradient magnitude threshold ($\tau_{m}$) ($\left|\nabla_{p}\right|\geq \tau_{m}$). For gradient direction of shadow pixels, we calculate the difference between them in current frame F and Reference frame *R* as presented below:

(3) $$\Delta \theta_{p}=arccos\left[\begin{array}{c} \frac{\nabla_{x}^{F}\nabla_{x}^{R}+\nabla_{y}^{F}\nabla_{y}^{R}}{{\left\{\begin{array}{c} \left(\nabla_{x}^{F^{2}}+\nabla_{y}^{F^{2}}\right)\left(\nabla_{x}^{R^{2}}+\nabla_{y}^{R^{2}}\right) \end{array}\right\}}^{1/2}}. \end{array}\right]$$

**Figure 3:**
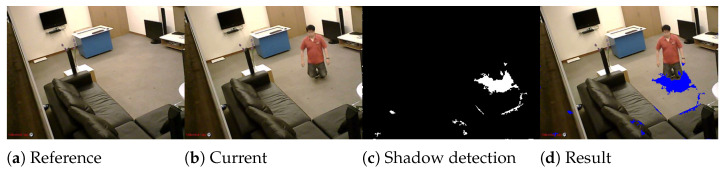
Illustration of Shadow detection. (**a**) The reference frame. (**b**) The current frame. (**c**) Shows the detected shadow pixels in current frame. (**d**) the result of shadow detection showed with blue color in current frame.

Then, we estimate the gradient direction correlation between current frame *F* and reference *R* at pixel p(i,j) in blob *B* by:

(4) $$C^{B}=\frac{1}{N}\sum_{p=1}^{N}H\left(\tau_{a}-\Delta \theta_{p}\right),$$

where *N* is the number of pixels in blob *B*, H(.) is a function which returns 1 if $\tau_{a}-\Delta \theta_{p}$ is positive or 0 otherwise; $\tau_{a}$ is gradient direction threshold; $C^{B}$ is the average gradient direction of pixels which are similar in frame *F* and reference *R*. All pixels in blob *B* are detected as shadow if the condition $C^{B}\geq \tau_{c}$ is satisfied, with $\tau_{c}$ is the correlation threshold. The values of thresholds $\tau_{m},\tau_{a},\tau_{c}$ are presented in Table 1. The shadow detection result of the proposed method is presented in Figure 3.

The foreground extracted by the CB method and shadow detection contains many blobs which form big and small objects. In order to identify the human silhouette from these blobs, we use two rules: (i) By using the OpenCV blob library [36], we remove all blobs which have a small area (i.e., <50 pixels), (ii) for big blobs, we classify them into many classes by using the rectangle distance, each class contains the nearest blobs using the Equation (5) (the minimal distance is defined empirically as 50 pixels).

(5) $$Distance\left(B1,B2\right)=min_{P1\in R1,P2\in R2}distance\left(P1,P2\right),$$

where B1, B2 correspond to blob 1 and blob 2; P1 and P2 are the closest point from rectangles R1 and R2 respectively. If the distance computed between two blobs is less than 50 pixels, then, they are in the same class, otherwise, each one is in a different class.

**Figure 4:**
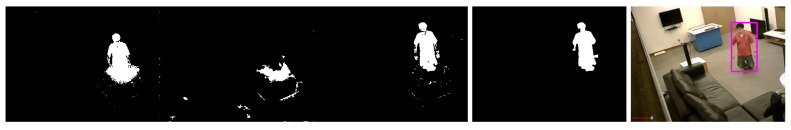
Human extraction result. First frame correspond to the Codebook result, the second one is the shadow detection. with simple subtraction between CB result and shadow detection, we get the third frame. After applying the second step from post processing, we get the result in the fourth frame. The last frame illustrates the tracked object correspond to the human silhouette.

Then, we determine the blob which corresponds to a human silhouette by using the motion of the blob’s pixels based on the optical flow result and the distance between the current and the previous positions of each class. Thus, the class which has a small distance and high motion is considered as the required class corresponding to the desired human silhouette. In Figure 4, we illustrate the human silhouette extraction from the background using our method where the last frame shows the required result.

### 3.2. Human Posture Representation

From literature, many approaches for human posture recognition have been proposed [17,18,19,37,38,39]. Generally, these methods can be divided into wearable sensors-based methods and computer vision-based methods. For the first category, the person needs to wear on his body some sensors or some kind of cloths that provides several features used to identify the person’s posture. Such kind of sensors is wearing a garment with strain sensors for recognizing the upper body posture [7], using trixial-accelerometer mounted on the waist of a person’s body in order to distinguish between human movement status [8]. Nevertheless, even if they have several advantages, these sensors need to be recharge or to change their power source periodically and it required to be carried by the elderly during his Activity of Daily Life (ADL), which conclude that these issues can be an inconvenience for them.

The second category of approaches consists of capturing images of the human body. Based on the image-processing techniques, variant features are extracted from the human shape and used for posture classification. In the literature, the central issue in shape analysis is to describe effectively the shape where its characteristics are a fundamental problem. In general, we can divide shape description techniques into two categories. The first one is contour-based methods [24,40,41,42] which analyze only the boundary information of the human body and using the matching techniques to discriminate between different shapes. However, the inconvenience of this method is that the interior information of the shape is ignored which can be resolved by the region-based methods such as in [18,43,44]. They take into account all information of the shape and analyze the interior contents. Such techniques are based on a projection histogram of the shape. In [18,43], the authors extract the histogram of the human shape using the centroid shape-context based on the Log-polar transform. Another technique uses Ellipse projection histogram as local features to describe the human shape [19].

Inspired by previous techniques, we propose a novel histogram projecting method to describe more correctly human shape in order to identify the human posture. The proposed projection histogram is based on the bounding box, where we divide our human shape into different partitions horizontally and vertically using several angles. The intersection of these partitions provides our projection histogram and it is considered as a shape descriptor with a powerful discriminative ability.

**Figure 5:**
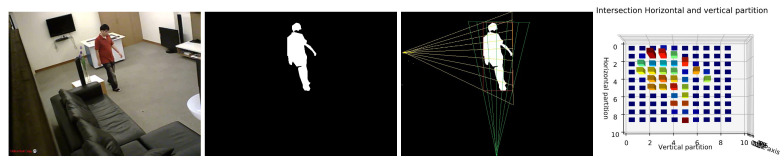
Illustration of the shape representation. First and second frames represent the original and human silhouette respectively. Third and fourth frame illustrate horizontal and vertical area partitions and corresponding histogram chart respectively.

After extracting the human silhouette from the background, we based on bounding box fitted on the human shape to extract our projection histogram as a human shape descriptor. Here, the reason for using a bounding box instead of an ellipse is that the ellipse does not take into account all the pixels of the shape. The comparison between them is presented in [45]. Depending on the rectangle center, its height and its width, we divide the human shape horizontally and vertically, as shown in Figure 5. We present below the whole Algorithm 1 which shows the different steps for computing the horizontal and vertical partition to extract the projection histogram as a descriptor of human posture.
**Algorithm 1:** Our proposed projection histogram algorithm1:**Input:** Number of partition *N*; Binary shape *S*; Height, Width of BB; Reference Horizontal Point ($P_{h}$); Reference Vertical Point ($P_{v}$)  2:**Output**: $\psi {\left(i,l\right)}_{N*N}$                                   ▹ Projection histogram3:1: **Initialization**:  4:       Number of partition *N*  5:       $\psi \left(i,l\right)\leftarrow 0;i,l=1,...,N;$  6:       $P_{v}\leftarrow \left(x_{v},y_{v}\right);$  7:        $P_{h}\leftarrow \left(x_{h},y_{h}\right);$  8:2: **Compute** $\theta_{H}$, $\theta_{V}$:  9:     $D_{H}=distance\left(P_{h},BB\right);$  10:     $D_{V}=distance\left(P_{v},BB\right);$  11:     $\theta_{H}=2*\arctan\left(\frac{Height*D_{H}}{2}\right);$                             ▹ Horizontal angle12:     $\theta_{V}=2*\arctan\left(\frac{Width*D_{V}}{2}\right);$                               ▹ Vertical angle13:     $\Delta \theta_{H}=\frac{\theta_{H}}{N};$                                  ▹ Horizontal partition step14:     $\Delta \theta_{V}=\frac{\theta_{V}}{N};$                                    ▹ Vertical partition step15:3: Let $A\leftarrow \{\left(x_{t},y_{t}\right),t=1,...,M\}$ be a Set of pixels in shape *S* and *M* is the total points.  16:4:**Loop for all points**  17:**For** t = 1 **to** *M* **Do**  18:  **if** $x_{h}-x_{t}\geq 0$**then**                           ▹ *Find pixel id of Horizontal partition19:        ${\left(\theta_{H}\right)}_{t}=\arctan\left(\frac{x_{h}-x_{t}}{y_{t}-y_{h}}\right);$  20:        $i=Round\left(\frac{{\left(\theta_{H}\right)}_{t}-\frac{\theta_{H}}{2}}{\Delta \theta_{H}}\right);$ 21:  **else** 22:        ${\left(\theta_{H}\right)}_{t}=\arctan\left(\frac{x_{t}-x_{h}}{y_{t}-y_{h}}\right);$  23:        $i=Round\left(\frac{{\left(\theta_{H}\right)}_{t}+\frac{\theta_{H}}{2}}{\Delta \theta_{H}}\right);$ 24:  **endif**  25:  **if** $y_{v}-y_{t}\geq 0$**then**                            ▹ *Find pixel id of vertical partition26:        ${\left(\theta_{V}\right)}_{t}=\arctan\left(\frac{y_{v}-y_{t}}{x_{t}-x_{v}}\right);$  27:        $l=Round\left(\frac{{\left(\theta_{V}\right)}_{t}-\frac{\theta_{V}}{2}}{\Delta \theta_{V}}\right);$ 28:  **else** 29:        ${\left(\theta_{V}\right)}_{t}=\arctan\left(\frac{y_{v}-y_{t}}{x_{t}-x_{v}}\right);$  30:        $l=Round\left(\frac{{\left(\theta_{V}\right)}_{t}+\frac{\theta_{V}}{2}}{\Delta \theta_{V}}\right);$  31:  **endif**  32:  $\psi \left(i,l\right)\leftarrow \psi \left(i,l\right)+1;$  33:**endFor** 34:**Return**$\psi \left(i,l\right);$

The output of Algorithm 1 is $\psi {\left(i,l\right)}_{N*N}$. The histogram is followed by normalisation using Equation (6). The goal of normalization is to make sure that the extracted histogram is invariant according to the human size and the distance from the camera.

(6) $$\hat{\psi }{\left(i,l\right)}_{N*N}=\frac{1}{M}\sum_{i}^{N}\sum_{j}^{N}\psi \left(i,l\right).$$

For experimental study, the number of partitions will be fixed to *N* = 10. With consideration of $C_{BB}\left(x_{c},y_{c}\right)$ as a center of bounding box and *H* as the height of the image, the Reference vertical point ($P_{h}$) and Reference horizontal point ($P_{v}$) are fixed to $\left(x_{h}=x_{c},y_{h}=0\right)$ and $\left(x_{v}=H,y_{v}=y_{c}\right)$.

By considering only local features to describe all human postures is not sufficient, where some similar postures are very difficult to differentiate between them, which leads the classifier to be confused. For this purpose, we add global features such as the horizontal and vertical angles ($\theta_{H}$, $\theta_{V}$) and the ratio between them ($\frac{\theta_{H}}{\theta_{V}}$). Then, we combine local feature and global feature as a whole vector feature for classification. The classification results of these two kinds of features will be experimentally discussed in Section 4.2.3.

The final dimension of the feature vector is N∗N + 3, where N∗N represents the local feature vector and 3 represents the three global features. The whole feature vector of the shape ($F_{S}$) is defined as follow:

(7) $$F_{S}=\left[\hat{\psi }{\left(i,l\right)}_{N*N},\theta_{V},\theta_{H},\frac{\theta_{H}}{\theta_{V}}\right].$$

### 3.3. Posture Classification

**Figure 6:**
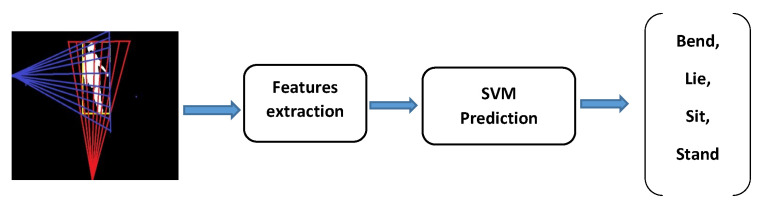
Posture classification.

The classifier can be used to attribute our vector feature $F_{S}$ (See Equation (7)) to one of the four posture categories (Lying Siting, Standing, and Bending) which are considered as fall-related posture. The classifier is employed to find a function which maps each feature vector into corresponding label space $Y_{k}=\left\{1,2,3,4\right\}$, where 1, 2, 3 and 4 represent bend, lie, sit and stand posture respectively. As we have several labels, we adopt a multiclass classifier for posture classification instead of a binary classifier. The whole operation is presented in Figure 6.

### 3.4. Fall Detection Rules

After the posture classification step, we check the existence of the fall when the output of the classifier is abnormal posture (lay or bend). For this purpose, we use the Algorithm 2 which is composed of four rules. In the first rule, we check if the posture is classified as “lie” or “bend”. Then, we verify if the posture is inside the floor which means if the percentage area of the human silhouette is high than a defined threshold. The value of the threshold is set to 85% and it is founded experimentally. For the posture transition, as most fall activities begin with stand posture and end to lay posture or begin with sit posture and end to lay posture. The time passing for a fall is an average of 20 frames, which is defined based on our experiments. Finally, if these above conditions are kept at a certain time with no motion, which exceeds the defined threshold (we use 25 frames), we confirm the fall detection and an alarm signal for help is triggered.

Floor information is crucial for fall confirmation because the fall activity always ends in lying posture on the floor. Many previous works incorporated the information of ground plane and showed good results [19,46,47].

By the reason of using only RGB camera for fall detection instead of using depth-cameras, and the datasets have been realized with different floor textures. We proposed to use manual segmentation to extract ground regions instead of using any supervised methods such as presented in [48] in order to evaluate our fall detection algorithm. We use only the first frame from any sequence video to extract the floor pixels.

**Figure 7:**
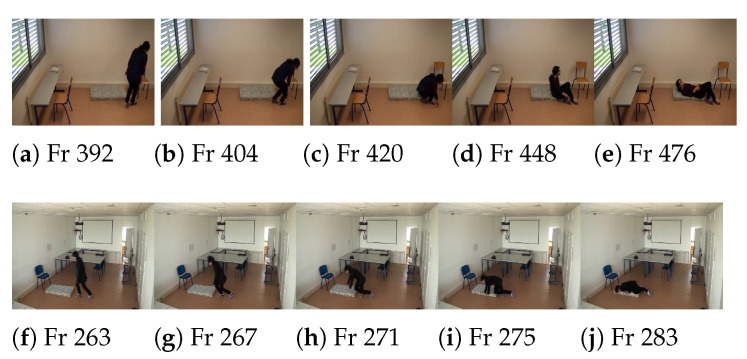
Illustration of posture transition frames of fall activity and normal lying activity. Frames (**a**–**e**) present a sequence of the lying activity. The lying activity starts from frame number Fr404 (**a**) and ends to frame number Fr476 (**e**). Frames (**f**–**j**) present sequence of the fall activity. The fall activity starts from frame number Fr263 (**f**) and endqws to frame number Fr283 (**j**).

To distinguish between similar activities as falling and lying, we based on time transition from stand to lay postures as shown in Figure 7. From this figure, we can see that the lying activity takes more than 80 frames (e.g., more than 3 s). However, for the fall activity, the number of frames for posture transition is less than 25 frames which means less than 1 second, and this is normal because the fall is an uncontrolled movement and the lying is a controlled movement. For performing this time transition, if the person’s posture is classified as a bend or lay and most of his body region is inside the floor, we count the number of frames between the current frame and the previous frame where the person is classified as a standing or sitting posture. If the number of frames is less than 25 frames, then, we return true as this activity may be considered as fall if the person stays inactivity for a while.
**Algorithm 2:** Fall detection strategy1:**Input:** Human Posture, Body area, inactivity time threshold T  2:**Output**: Fall or no-Fall  3:**Repeat**:  4:       CheckAbnormalPosture()  5:       CheckInsideFloor(Area)  6:       TransitionPosture()  7:       CheckInactivityTime (T)  8:       **if** the conditions 4, 5, 6 and 7 are True **then** return Fall  9:       **Else** go to step 3

## 4. Experiments Results and Discussion

This section shows the performance of the proposed fall detection system. The architecture has been implemented using the C++ language, Microsoft Visual Studio Express 2012 and OpenCV library 2.4.13 for Background subtraction, Feature extraction, and classification step. The experiments (training, testing) were carried out on a Notebook with Intel(R) Core(TM) i7-6700HQ CPU and 2.60 Hz and 12.00 GB of RAM. With intensively, we conduct on a different datasets where we split them into two categories; the posture recognition dataset and fall activities dataset. More details are presented in the next section.

### 4.1. Background Subtraction

**Figure 8:**
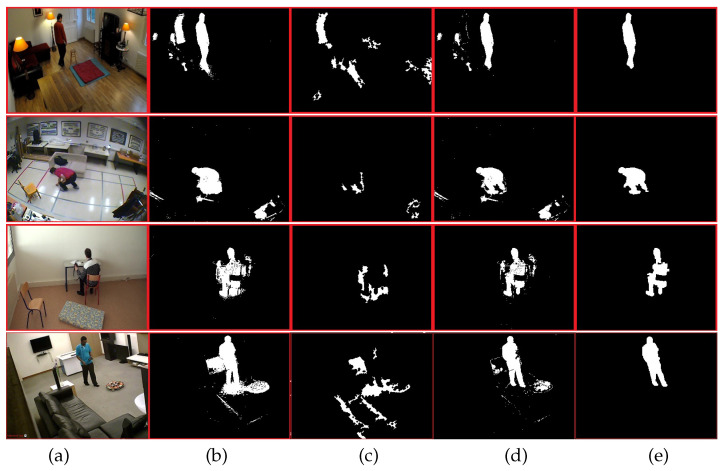
Backgroun subtraction results. (**a**) original frame, (**b**) codebook algorithm result, (**c**) shadow detection, (**d**) difference between image (**b**) and image (**c**). (**e**) The human silhouette detection after applying simple morphological operation.

In this part, we show the performance of the background subtraction algorithm. Some significant results are illustrated in Figure 8. The biggest drawbacks of any method based on background subtraction are lighting change and shadows. For this purpose, we have separately processed the shadow detection and background subtraction. By using the result of shadow detection, we subtract it from the CB algorithm result, followed by morphological operation for removing small blobs. The last column in Figure 8 represents our human silhouette extraction result.

As we can see from Figure 8, the condition of light is different in all original images (a). The person can walk near an object as shown in the last row and the person can do his daily activity life with different postures. Under this conditions, the human silhouette is correctly extracted from the background.

### 4.2. Posture Classification

We perform our posture classification based on the library LIBSVM using SVM classifiers [49]. We use C-SVM multiclass classifier with a kernel of Radial Basis Function (RBF). The default parameter was used except gamma (g) of RBF and the cost (c) of C-SVM which were modified as 0.01 and 100 respectively.

The performance of our method is conducted on datasets described in the next section where four main experiments were made including features evaluation, number of partitions evaluation, comparison of features extraction methods from state of the art and classifiers comparison.

#### 4.2.1. Postures Datasets

To evaluate our posture recognition method and to compare it with other methods, we use two posture datasests which are composed of 2D human silhouettes, including lying, standing, siting and bending posture.

**Figure 9:**
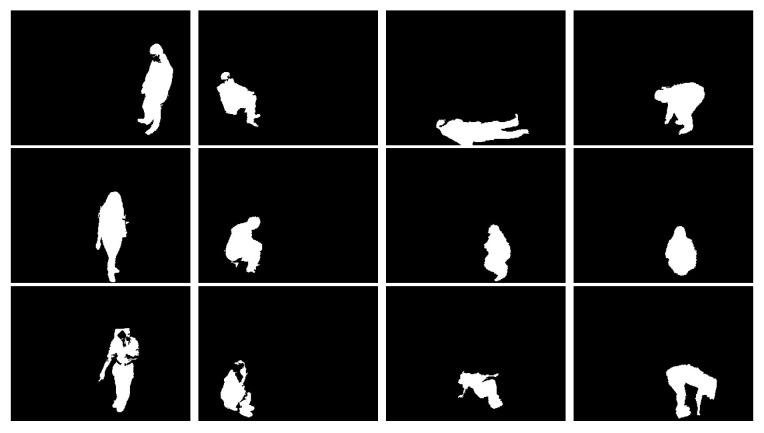
Posture Samples from Dataset D1. Column 1, 2, 3 and column 4 correspond to Standing, Siting, lying and Bending postures respectively.

Dataset (D1) [17]: This dataset was released using a single RGB camera. 10 people were invited to participated as volunteers for the experiments simulating. Each person was asked to simulate postures in different directions so that the constructed classifier should be robust to view angles. The postures of each person *i* are stored in folder named $P_{i}$ with i=1,...,10. The whole dataset contains 3216 postures including 810 lies, 804 stands, 833 bends and 769 sits. Some keyframes are shown in Figure 9.

**Figure 10:**
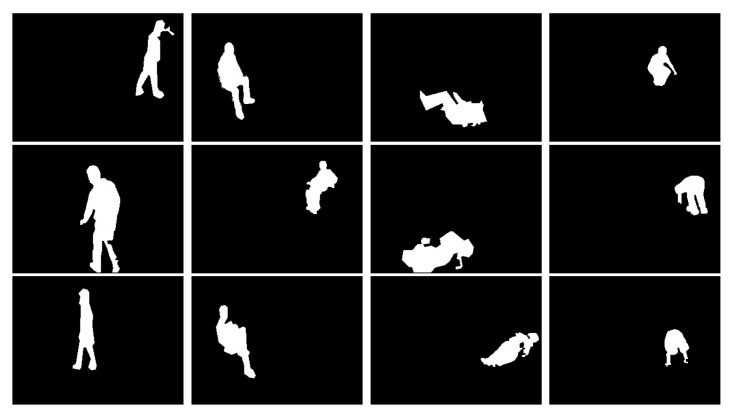
Posture samples from the Dataset D2. Columns 1–4 correspond to standing, sitting, lying and bending postures, respectively.

Dataset (D2): based on dataset [10,50], we released our posture dataset using the background subtraction algorithm [34] to extract the human silhouette from the videos. This dataset is composed of 2865 postures includes 444 sits, 1394 lies, 453 bends and 576 stands. Figure 10 shows some keyframes from this dataset.

In Table 2, we show the description of some characteristics and difficulties challenge of both datasets.

#### 4.2.2. Performance Criteria

To evaluate the efficiency of our proposed method, there are several indicators based on True Positive (TP), False Positive (FP), True Negative (TN) and False Negative (FN) as shown in Table 3.

In this paper, we use the accuracy (Acc) as single indicator which is the percentage of items classified correctly. It is the most straightforward measure of classifier quality.

(8) $$Acc=\frac{TP+TN}{TP+FP+TN+FN}$$

The precision indicator is the number of items correctly identified as positive out of total items identified as positive. It gives us information about a classifier’s performance with respect to false positives (how did we caught).

(9) $$Prec=\frac{TP}{TP+FN},Recall=\frac{TP}{TP+FP}.$$

Recall is also an other indicator. It is the number of items correctly identified as positive out of total true positives. It gives us information about a classifier’s performance with respect to false negatives( how many did we miss).

The traditional F-measure or balanced F-Score is the harmonic mean of precision and recall:

(10) $$F-Score=\frac{2\times Precision\times Recall}{Precision+Recall}$$

#### 4.2.3. Local and Global Features Comparison

We first compare between the global and local features. The common 10-fold cross-validation [51] was used to evaluate the features.

As shown in Table 4, we compare the classification result obtained when we use the local feature or global feature alone and when we use the combination between them. The SVM is applied for classification and from this table, we can see that the combined feature provides a high accuracy than using either feature alone.

Table 5 and Table 6 illustrate the performance metrics for the proposed method applied to datasets D1 and D2 using the combined features. The precision, recall and F-Score are the selected evaluation metrics for posture classification which are computed based on the confusion matrix. From these tables, all postures are well classified with high values for all metrics.

#### 4.2.4. Proposed Projection Histogram Evaluation

**Figure 11:**
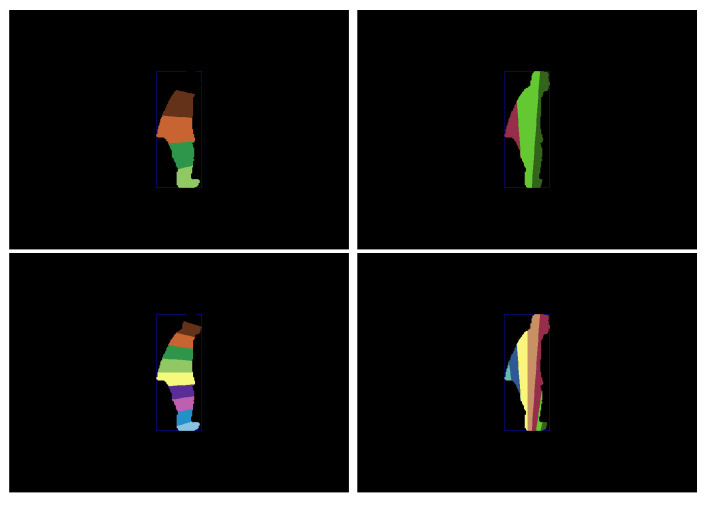
Illustration of the number of partitions horizontally and vertically of human shape. The first row presents the human shape with 5 partitions. The second row presents 10 partitions.

Deciding the number of partition which can be efficient for posture classification is difficult as shown in Figure 11.

**Figure 12:**
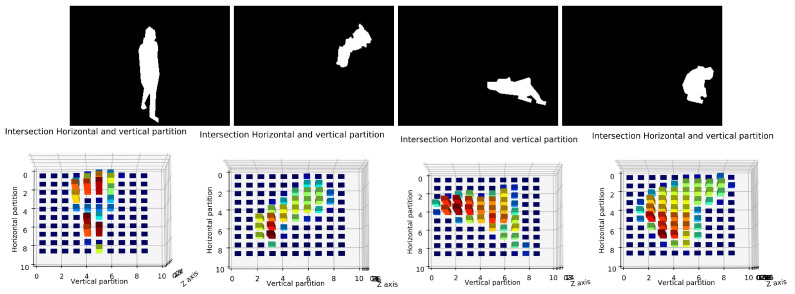
Illustration of human body posture and his projection histogram. First row presents the human silhouette postures which are standing, sitting, lying and bending postures. Second row notices the correspond projection histogram which are the intersection between horizontal and vertical partition.

Therefore, we used several numbers of partitions and we tested each one by using the dataset D1 as shown in Table 7. We used 5 partitions (bins), 10, 15 and 20 partitions. As we can see from this table, the 10 partitions give high accuracy than others for the most of cases. These results can be justified that if the number of partitions is small, we lose a lot of information where we got a large number of instances (pixels) in each bin which could be similar between postures. For a large number of partitions, we got several partitions (bins) with empty instances or with equal instances between different postures, which leads to the difficulties in distinguishing between them. As results, we considered 10 partitions as the default value of our system in all next experiments. Figure 12 shows an example of four projection histograms with 10 partition for stand, sit, lay and bend postures.

#### 4.2.5. Features Extraction Methods Comparison

To show the performance of our feature extraction method for posture recognition. We compare our method with CNN [31], ellipse descriptor [19] and shape context [52] using Dataset D1 and Dataset D2. Table 8 refers to result using D1 and Table 9 refers to result using D2.

In Table 8, we used each particular individual ($P_{i}$) postures for testing, the other posture are used for training. Our method shows significant advantages compared to other methods where it outperforms ellipse descriptor and shape context methods. For the majority of individuals cases with a higher accuracy. Compared to the CNN method, even the deep learning has a high performance in compared to basically methods, our method gives good results where it outperforms for several cases.

In Table 9, we illustrate the comparison result obtained using the dataset D2 where we choose 70% of data for training and 30% for testing. The whole dataset D2 contains 1865 postures. The results show that our method achieve high accuracy than others methods and low in false detection.

An other comparison is performed as shown in Table 10. We compare the performance achieved by our approach and some existing approaches of features extraction discussed in the state of the arts using the same dataset. From this table, the proposed approach presents favorable results compared to those achieved by the ellipse descriptor. The projection histogram [19] is based only on ellipse which does not take into account all pixels in the human silhouette. For the silhouette area method [53], the variations of the human silhouette area are view-invariant, but it is widely depended on the background update strategy. The bounding box ratio method [54] is based only on global features and it is very easy to implement. The normalized directional histogram [20] is used to derive static and dynamic features based on the ellipse.

#### 4.2.6. Classifiers Comparison

**Figure 13:**
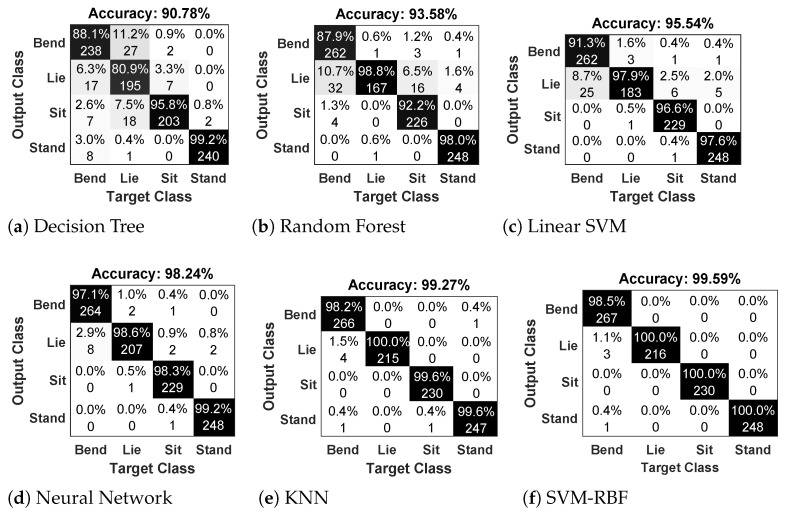
Confusion matrix of posture classification. Figures (**a**–**f**) show the result obtained using classifiers Decision Tree, Random Forest, Linear SVM, Neural Netword, KNN and SVM-RBF respectively.

In this section, we compare the performance of the proposed method with results from some common used machine learning algorithms, including Linear SVM (L-SVM), Random Forest (RF), Decision tree (DT), K-Nearest Neigbor (K-NN), Neural Network (NN), and SVM-RBF. Figure 13 presents the confusion matrix results using Dataset D1. From this figure, we can see that the SVM-RBF classifier gives the highest accuracy compared to others classifiers for all types of postures.

### 4.3. Fall Detection

#### 4.3.1. Fall Datasets

**Figure 14:**
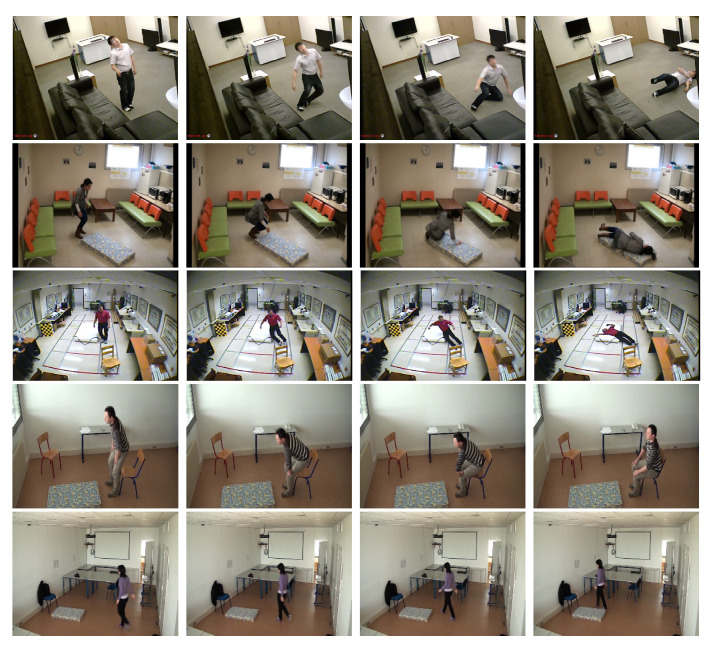
Key frames from several datasets. The fall and normal activities. First row, second and third row show the fall activities from Dataset D3, D4 and D5 respectively. The Fourth and the last row show the sitting and walking activity from the Dataset D2.

For fall detection system evaluation, we use three available datasets released in different locations using different types of cameras. Some keyframes are shown in Figure 14. More details of each dataset are presented below.

Dataset (D3) [10]: This dataset was released using single RGB camera in one room. The camera was positioned on the ceiling of the room. Different volunteers participated to simulate several activities as walk, sit, fall, crouching-down, lay and bending. It contains 20 videos with 45 normal activities and 29 falls. Some keyframes are

Dataset (D4) [50]: This dataset was recorded on four different rooms (Office, Coffee room, Home, Lecture room) by using a single RGB camera. The camera is positioned 2 m high from the floor. Several activities has been simulated by different volunteers including walks, sits, falls, etc. The dataset is composed of 249 videos. About 57 of these contained normal activities and 192 contained a fall incident.

Dataset (D5) [22]: This dataset was recorded by eight calibrated RGB cameras. It is composed of 24 scenarios. All activities were simulated by one volunteer. There are simulation of falls and normal daily activities. In each, the person engages in many activities, such as falling, sitting, walking, sitting and lying on the sofa.

#### 4.3.2. Experiment Fall Recognition Results

**Figure 15:**
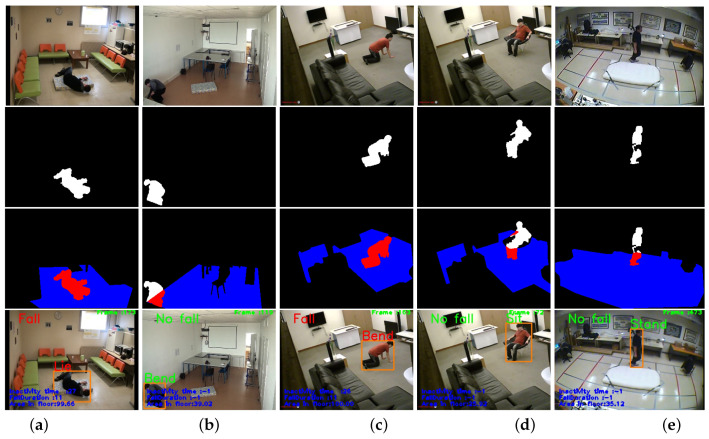
Illustration of fall, bend, sit, and stand activities results. Columns (**a**–**e**) represent fall, bending, sitting and standing activities, respectively. Rows 1–4, from top to bottom, represent reference images, human silhouettes, human silhouette with floor intersection result, and the result of our fall detection system respectively.

For fall detection, according to the Algorithm 2 in Section 3.4, the posture classification along with the detection floor information are used to detect falls. In Figure 15, we show some human posture classification results with floor information. The frame (a) shows a person who has fallen on the floor, the ‘lying’ posture is detected and the most body region is inside the ground region, if this posture is a result of an activity that is started by standing or siting posture and the number of frames of this transition is less than 25 frames (fall duration = 11), then the fall is detected if this posture kept inactivity for certain time which exceed a defined threshold (threshold = 1 s). The bend posture is shown in figure (b), where the body region is not completely inside the ground region, which confirms that it is a normal activity. An other case is shown in frame (c), the posture is classified as bend and the posture time transition is less than threshold, while this posture kept immobile for more than threshold, the system has classified it as fall activity. The posture in frame (d) is classified as a sitting posture, so while this posture is considered normal activity, our system continue to operate normally until detecting some abnormal postures. The frame (e) shows a person who is standing/walking on the floor and the system detects it as normal activity.

#### 4.3.3. Fall Detection System

To evaluate our fall detection system, we collect several fall videos from Datasets D3–D5. Some videos from these datasets are not considered because of three reasons in which our method cannot be performed; (i) some videos are very short and there is no additional time for fall confirmation, (ii) the first frame in videos contains the person which confused with our background subtraction algorithm that based on first 50 frames to construct the background model, and also with floor extraction. (iii) The person is behind an object when he falls in which his shape is mostly not visible to the camera.

As shown in Table 11, the total number of fall activities is 159, and 136 for non-fall activities. In order to use posture classification of person for fall detection, the postures from dataset D1 and dataset D2 are combined and used to train the SVM classifier. From this table, we can see that our system can detect 155 as falls out of 159 (97.48%) fall activities, while for non-fall activities, only four are detected as falls out of 136 (2.94%); those errors can be justified by the reason when the person is bending to the floor for a while with no motion which exceeds the thresholds, the system detects it as a fall.

In Table 12, we show the experiments result of the proposed method and other methods in the state of the art. As we can see, our method achieves a high accuracy than other methods. The proposed method achieves less recall value compared to the methods [26,27], while it is higher in precision. That means that our method is great in ability of fall detection, while it still needs to be improved to reduce the misclassification of normal activities. Compared to the method [30], our method is better in reducing the false alarm.

As result, the experiment result obtained are acceptable and it proves the effectiveness of our method for fall recognition.

### 4.4. Discussion

The proposed approach is using posture recognition for a fall detection system. The proposed system is convenient while the elderly do not need to carry any sensors on their body which are generally affected by background noise in the environment. The proposed descriptor for posture classification provides a high rate of classification compared to other posture recognition methods. With a large dataset, including different human postures captured with different cameras, the classifier can effectively distinguish different types of postures. The procedure is totally automatic and there is no need to set up any thresholds to distinguish between them. It is fast and efficient where we based only on a single frame to extract and perform the posture classification instead of using a set of frames as the video clip-based methods [55,56]. Moreover, we use only traditional classifier instead of using deep-learning methods such as in [17,31] which are high in computational time.

Compared to the state-of-the-art method, in posture classification, our proposed approach has the following characteristics, which are the novelties of our work:

As the main component of fall detection is based on the human posture classification, the human extraction from the background should be robust and efficient. For this purpose, we use an effective background subtraction combined with shadow detection in order to cope with background change such as light and shadow problems. In each frame, the blob operations are used to select the not required objects which are classified as furniture or ghost and put them into the background model. Hence, in the next frame, they will disappear from the foreground.

A new human silhouette descriptor is used for posture classification which is more robust compared to the Ellipse-based projection histogram and CNN method. Fall confirmation required floor information in order to distinguish between a human lying on the sofa and a human falling. Most error detection in the previous systems refers to this problem. So, as a solution, confirmation using the floor information reduces the error detection.

However, some problems still occur in our system such as in [10,18,20]. Our system is designed for monitoring a single person living alone at home, which is not adequate for some special cases. Such cases include the presence of multiple people in the home and when the elderly have a large size pet (near to the camera) or small enough (far from the camera). If there is more than one person at home, it is not necessary for our system to operate, as the other persons can ask for help if the elderly person falls. The system will be automatically turned off and sleep until the elderly turns on manually or based on some techniques for counting the number of people, such as [57,58]. For the case where the elderly are large or small enough, the human silhouette is the only one extracted from the foreground, whether to determine if it is a pet or human silhouette, there are some object classification techniques such as [59,60,61], or using deep learning methods as in [62].

Another problem is the occlusion. The home environment often contains many objects as tables, sofa, chairs, and other objects. These objects sometimes cause occlusion which occurs when the elderly is behind one of them. As result, it deteriorates the performance of the fall detection system. For this purpose, adding more than one camera for monitoring the elderly can be used to make sure that the whole elderly body or most of his body is in front of at least one camera. For fall detection, each camera performs the fall detection and results are combined using some techniques such as majority voting to make a decision. Such strategies of majority voting were used in [24].

## 5. Conclusions

In this work, we have proposed a fall detection system for elderly people based on posture recognition using a single camera. The process of our new approach is straightforward. Indeed, in our system, we first subtract the background to extract the human silhouette from the frame video using the CodeBook algorithm. A shadow detection was added to improve the foreground detection. Then, we are using our proposed method to extract local and global features from this human silhouette. These features are combined and fed up to the classifier to predict his posture type such as lay, sit, stand and bend posture. The local features are the projection histogram extracted based on the bounding box; the human silhouette is divided horizontally and vertically into equal partitions using an angle step. The intersection of horizontal and vertical partitions provides our local feature. The global features are the horizontal and vertical angles combined with the ratio between them. The evaluation of the proposed features extracted for posture recognition shows a good result compared to the state-of-the-art methods. Furthermore, we evaluate our method using the common classifiers including SVM, NN, KNN, RF and DT. Experimental results showed that the SVM classifier is an interesting classifier. After posture classification, four rules for fall detection are used. The fall is detected if these conditions are satisfied. The experiment results show that our fall detection system obtains better performance with high accuracy in fall recognition and low in false detection. Although these results, we still have some problems as discussed above. The multi-person and occlusions are two drawbacks of our system. As future work, we plan to use our proposed approach using at least two cameras by adding people counting techniques and object recognition.

## Figures and Tables

**Table 1:** Description and values of parameters threshold.

Threshold	Description	Range	Values
α	lower threshold for V channel	[0,1)	0.21
β	upper threshold for V channel	[0,1)	0.99
$\tau_{H}$	*H* channel threshold	[0,255]	93
$\tau_{S}$	*S* channel threshold	[0,255]	76
$\tau_{m}$	Gradient magnitude threshold used to assigned each pixel p(i,j) as shadow or foreground	[0,10]	6
$\tau_{a}$	Gradient magnitude coherence threshold used to assigned each pixel in blob as shadow or foreground	[0,π]	$\frac{\pi }{10}$
$\tau_{c}$	Correlation threshold used to assigned each gradient directions blob B as shadow region or foreground	[0,1]	0.2

**Table 2:** Characteristics challenge of each dataset.

Dataset	Person	Local	Human Shape Dimension	Angle of View	Ambiguity Postures
D1	man, woman	one local	big, small	several	yes
D2	man, women	5 locals	big, small	several	yes

**Table 3:** Confusion matrix of classification result.

		Actual		
		**Positive**	**Negative**	**Total**
Predicted	Positive	TP	FP	TP+FP
	Negative	FN	TN	FN+TN
	Total	TP+FN	FP+TN	*N*

**Table 4:** Results features comparison for both datasets D1 and D2.

Dataset		Global	Local	Both
**D1**	Accuracy	90.06%	97.00%	99.90%
	False Detection	6.5433%	0.0625%	0.0401 %
**D2**	Accuracy	89.63%	98%	99.84%
	False Detection	0.6303%	0.10%	0.0746%

**Table 5:** Postures classification for dataset D1.

Postures	Precision	Recall	F-Score	♯train	♯test
Bend	99%	100%	99.49%	566	267
Lie	100%	99%	99.49%	591	219
Sit	100%	100%	100%	539	230
Stand	100%	100%	100%	555	249

**Table 6:** Postures classification for dataset D2.

Postures	Precision	Recall	F-Score	♯train	♯test
Bend	100%	99%	99.49%	309	137
Lie	100%	100%	100%	978	406
Sit	99%	100%	99.49%	320	118
Stand	100%	99%	99.49%	374	188

**Table 7:** The number of partition evaluation for features extraction.

Number of Partition	P1	P2	P3	P4	P5	P6	P7	P8	P9	P10
5	94.68%	90.71%	97.81%	91.93%	89.37%	90.85%	89.11%	**97.53**%	91.86%	90.12%
10	**95.62**%	**96.90**%	**99.06**%	**96.77**%	89.37%	**94.39**%	89.70%	96.92%	**98.30**%	92.59%
15	**95.62**%	**96.90**%	**99.06**%	95.16%	**91.56**%	92.62%	92.35%	96.61%	97.96%	92.59%
20	**95.62**%	94.11%	**99.06** %	95.16%	90.62%	89.97%	**92.64**%	96.61%	95.25%	**93.20**%

**Table 8:** Postures classification comparison using Dataset D1.

Method	P1	P2	P3	P4	P5	P6	P7	P8	P9	P10
CNN [31]	**96.88%**	**97.83%**	96.56%	92.58%	**95.31%**	92.04%	**93.24%**	96.62%	93.56%	**94.75**%
Shape Context [52]	92.18%	81.11%	88.12%	89.35%	85.93%	78.17%	79.41%	88.00%	83.72%	88.27%
Ellipse descriptor [19]	74.03%	67.93%	66.34%	71.85%	66.66%	66.46%	73.49%	72.38%	63.76%	71.65%
Our method	95.62%	96.90%	**99.06**%	**96.77**%	89.37%	**94.40%**	89.70%	**96.92**%	**98.30**%	92.59%

**Table 9:** Postures classification accuracy comparison of different features- extraction methods using Dataset D2.

Method	Accuracy	False Detection Rate
Shape Context [52]	98.37%	2.0%
Ellipse descriptor [19]	97.05%	3.5%
Our Method	**99.78%**	**0.5%**

**Table 10:** Comparison of different features extraction methods.

Method	Accuracy (%)
Ellipse descriptor [19]	96.1
Silhouette area [53]	95.2
Bounding Box ratio [54]	82.2
normalized directional histogram [20]	97.1
Our method	**99.90**

**Table 11:** Results of fall detection system.

	♯Activities	Detected as Falls	Detected as Non-Fall
Falls	159	155	4
Walking/Standing	40	0	40
Sitting	51	0	51
Bending	20	2	18
Lying	25	2	23

**Table 12:** Results of fall detection system.

Methods	Accuracy (%)	Precision(%)	Recall(%)
Chua [10]	93.30	-	-
Rhuma et al. [19]	96.09	-	-
Harrou et al. [26]	96.66	94.00	**100**
Wang et al. [30]	96.91	**97.64**	96.51
Jeffin et al. [27]	97.14	93.75	**100**
Our Method	**97.29**	97.48	97.48

## Data Availability

No new data were created or analyzed in this study. Data sharing is not applicable to this article.
